# Poly[diaqua-1κ^2^ *O*-bis[μ_3_-2-(1*H*-tetra­zol-5-yl)benzoato(2−)]dicadmium(II)]

**DOI:** 10.1107/S1600536808020503

**Published:** 2008-07-09

**Authors:** Xiu-Zhi Li, Bao-Zhen Wu, Zhi-Rong Qu

**Affiliations:** a Ordered Matter Science Research Center, College of Chemistry and Chemical Engineering, Southeast University, Nanjing 210096, People’s Republic of China

## Abstract

The title compound, [Cd_2_(C_8_H_4_N_4_O_2_)_2_(H_2_O)_2_]_*n*_, is a coordination polymer prepared by the hydro­thermal reaction of cadmium(II) chloride and 2-(1*H*-tetra­zol-5-yl)benzoic acid. Two types of coordinated cadmium cations exist in the structure. One is located on a twofold axis and is coordinated by four O and two N atoms from four symmetry-related ligands, forming a trigonal-prismatic coordination polyhedron. The other is located on an inversion center and is octa­hedrally coordinated by two N and two O atoms from two ligands in equatorial sites, and two water mol­ecules in axial sites. The organic ligand bridges three Cd atoms, through a carboxyl­ate group and two N atoms of the tetra­zolate unit. This mode of coordination results in a two-dimensional framework. The crystal structure is stabilized by inter­molecular O—H⋯O and O—H⋯N hydrogen bonds.

## Related literature

For the chemistry of tetra­zole derivatives, see: Xiong *et al.* (2002 ▶); Xue *et al.* (2002 ▶); Dunica *et al.* (1991 ▶); Wang *et al.* (2005 ▶); Wittenberger *et al.* (1993 ▶); Hu *et al.* (2007 ▶).
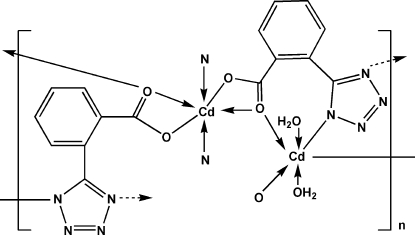


## Experimental

### 

#### Crystal data

[Cd_2_(C_8_H_4_N_4_O_2_)_2_(H_2_O)_2_]
*M* *_r_* = 637.16
Monoclinic, 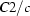

*a* = 19.886 (4) Å
*b* = 7.3522 (15) Å
*c* = 15.409 (3) Å
β = 115.97 (3)°
*V* = 2025.4 (7) Å^3^
*Z* = 4
Mo *K*α radiation
μ = 2.15 mm^−1^
*T* = 293 (2) K
0.35 × 0.30 × 0.10 mm

#### Data collection

Rigaku SCXmini diffractometer
Absorption correction: multi-scan (*CrystalClear*; Rigaku, 2005 ▶) *T*_min_ = 0.473, *T*_max_ = 0.809
8913 measured reflections
1976 independent reflections
1922 reflections with *I* > 2σ(*I*)
*R*_int_ = 0.029

#### Refinement

*R*[*F* ^2^ > 2σ(*F* ^2^)] = 0.018
*wR*(*F* ^2^) = 0.046
*S* = 1.13
1976 reflections
156 parameters
H atoms treated by a mixture of independent and constrained refinement
Δρ_max_ = 0.36 e Å^−3^
Δρ_min_ = −0.42 e Å^−3^

### 

Data collection: *CrystalClear* (Rigaku, 2005 ▶); cell refinement: *CrystalClear*; data reduction: *CrystalClear*; program(s) used to solve structure: *SHELXS97* (Sheldrick, 2008 ▶); program(s) used to refine structure: *SHELXL97* (Sheldrick, 2008 ▶); molecular graphics: *SHELXTL* (Sheldrick, 2008 ▶); software used to prepare material for publication: *PRPKAPPA* (Ferguson, 1999 ▶).

## Supplementary Material

Crystal structure: contains datablocks I, global. DOI: 10.1107/S1600536808020503/bh2178sup1.cif

Structure factors: contains datablocks I. DOI: 10.1107/S1600536808020503/bh2178Isup2.hkl

Additional supplementary materials:  crystallographic information; 3D view; checkCIF report

## Figures and Tables

**Table 1:** Hydrogen-bond geometry (Å, °)

*D*—H⋯*A*	*D*—H	H⋯*A*	*D*⋯*A*	*D*—H⋯*A*
O1*W*—H1*W*⋯O2^i^	0.75 (4)	2.06 (4)	2.758 (2)	156 (4)
O1*W*—H2*W*⋯N2^ii^	0.87 (4)	2.14 (4)	2.961 (3)	155 (3)

Symmetry codes: (i) 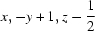; (ii) 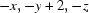.

**Fig. 1.**
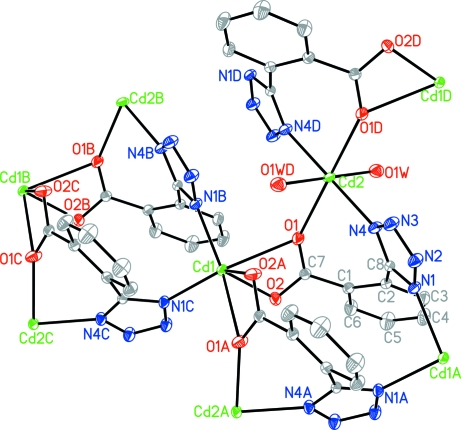
A partial packing diagram of the title compound, with displacement ellipsoids drawn at the 30% probability level. H atoms have been omitted for clarity. [Symmetry codes: (A) -x, -y + 1, -z; (B) -x, +y, 1/2 - z; (C) +x, +y - 1, +z; (D) -x, +y - 1, 1/2 - z.]

**Fig. 2.**
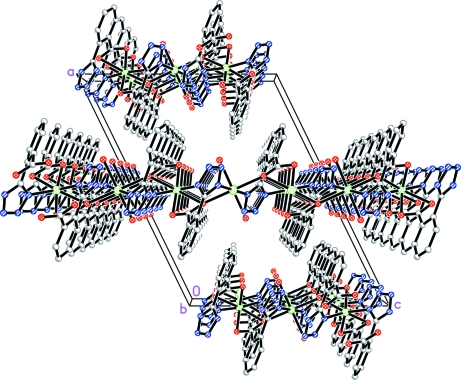
Packing diagram of the title compound, showing the structure along the b axis. No displacement parameters are displayed and H atoms have been omitted for clarity.

## supplementary crystallographic information

### Comment

Coordination frameworks have received much attention over the past decade because of their potential applications. Multifunctional organic ligands are necessary to construct such frameworks. 2-(1*H*-tetrazol-5-yl)benzoic acid is a ligand with two functional groups, one carboxylate group and one tetrazole ring. Tetrazole compounds have a wide range of applications in coordination chemistry, medicinal chemistry and material science (Hu *et al.*, 2007; Xiong *et al.*, 2002; Xue *et al.*, 2002; Wang *et al.*, 2005; Dunica *et al.*, 1991; Wittenberger *et al.*, 1993). We report here the crystal structure of the title compound, which was obtained by the hydrothermal reaction of cadmium chloride and 2-(1*H*-tetrazol-5-yl)benzoic acid.

In the structure of this compound, two types of coordinated cadmium cations exist (Fig. 1). Cd1 is located on a 2-fold rotation axis, and is trigonal prismatically coordinated by two O and two N from four different ligands. While Cd2 is loacated on an inversion center, and is octahedrally coordinated by two N and two O from two ligands at equatorial sites, and two O atoms of H_2_O at axial sites. The organic ligand bridges three Cd atoms by coordinating one Cd atom through one N atom from the tetrazole unit, by coordinating the other Cd atom through one O atom and one µ_3_-O from the carboxylate unit, and by coordinating a third Cd atom through a N atom from the tetrazole unit and one µ_3_-O from the carboxylate unit. Such an arrangement makes Cd1 and Cd2 bridged by one µ_3_-O carboxylate group and the tetrazole unit, resulting in a two-dimensional framework (Fig. 2). The crystal structure is stabilized by intermolecular O—H···O and O—H···N hydrogen bonds (Table 1).

### Experimental

A mixture of CdCl_2_ (0.2 mmol) and 2-(1*H*-tetrazol-5-yl)benzoic acid (0.2 mmol) in H_2_O (4 ml) was heated in a Pyrex tube at 373 K for two days. After slowly cooling down to room temperature over a period of 12 h., colourless crystals of the title compound suitable for diffraction were isolated.

### Refinement

Water H atoms were found in a difference map and refined freely. Other H atoms positions were calculated geometrically and these H atoms were allowed to ride on their carrier C atoms with C—H = 0.93 Å, and *U*_iso_(H) = 1.2 *U*_eq_(C).

### Crystal data

**Table:**

[Cd_2_(C_8_H_4_N_4_O_2_)_2_(H_2_O)_2_]	*F*_000_ = 1232
*M**_r_* = 637.16	*D*_x_ = 2.090 Mg m^−^^3^
Monoclinic, *C*2/*c*	Mo *K*α radiation λ = 0.71073 Å
Hall symbol: -C 2yc	Cell parameters from 0 reflections
*a* = 19.886 (4) Å	θ = 3.1–27.5º
*b* = 7.3522 (15) Å	µ = 2.15 mm^−^^1^
*c* = 15.409 (3) Å	*T* = 293 (2) K
β = 115.97 (3)º	Block, colourless
*V* = 2025.4 (7) Å^3^	0.35 × 0.30 × 0.10 mm
*Z* = 4	

### Data collection

**Table:**

Rigaku SCXmini diffractometer	1976 independent reflections
Radiation source: fine-focus sealed tube	1922 reflections with *I* > 2σ(*I*)
Monochromator: graphite	*R*_int_ = 0.029
Detector resolution: 13.6612 pixels mm^-1^	θ_max_ = 26.0º
*T* = 293(2) K	θ_min_ = 3.0º
CCD Profile fitting scans	*h* = −24→24
Absorption correction: multi-scan(CrystalClear; Rigaku, 2005)	*k* = −9→9
*T*_min_ = 0.473, *T*_max_ = 0.809	*l* = −18→18
8913 measured reflections	

### Refinement

**Table:**

Refinement on *F*^2^	Hydrogen site location: inferred from neighbouring sites
Least-squares matrix: full	H atoms treated by a mixture of independent and constrained refinement
*R*[*F*^2^ > 2σ(*F*^2^)] = 0.018	*w* = 1/[σ^2^(*F*_o_^2^) + (0.0214*P*)^2^ + 3.1517*P*] where *P* = (*F*_o_^2^ + 2*F*_c_^2^)/3
*wR*(*F*^2^) = 0.046	(Δ/σ)_max_ = 0.001
*S* = 1.13	Δρ_max_ = 0.36 e Å^−^^3^
1976 reflections	Δρ_min_ = −0.42 e Å^−^^3^
156 parameters	Extinction correction: SHELXL97 (Sheldrick, 2008), Fc^*^=kFc[1+0.001xFc^2^λ^3^/sin(2θ)]^-1/4^
Primary atom site location: structure-invariant direct methods	Extinction coefficient: 0.00235 (14)
Secondary atom site location: difference Fourier map	

### Fractional atomic coordinates and isotropic or equivalent isotropic displacement parameters (Å^2^)

**Table:**

	*x*	*y*	*z*	*U*_iso_*/*U*_eq_	
Cd1	0.0000	0.21274 (3)	0.2500	0.02355 (9)	
Cd2	0.0000	0.5000	0.0000	0.02567 (9)	
O1	0.05131 (10)	0.4374 (2)	0.16197 (11)	0.0376 (4)	
O1W	0.11318 (10)	0.6113 (3)	0.00533 (13)	0.0352 (4)	
H1W	0.115 (2)	0.579 (5)	−0.040 (3)	0.063 (11)*	
H2W	0.115 (2)	0.730 (6)	0.006 (3)	0.071 (11)*	
O2	0.09395 (10)	0.4136 (2)	0.31727 (11)	0.0361 (4)	
N1	−0.01798 (10)	1.0095 (2)	0.13491 (13)	0.0239 (4)	
N2	−0.08259 (11)	1.0024 (2)	0.05335 (14)	0.0303 (4)	
N3	−0.08190 (11)	0.8588 (3)	0.00480 (13)	0.0319 (4)	
N4	−0.01705 (10)	0.7689 (2)	0.05357 (13)	0.0264 (4)	
C1	0.12913 (12)	0.6784 (3)	0.26098 (15)	0.0235 (4)	
C2	0.09950 (11)	0.8385 (3)	0.20847 (14)	0.0223 (4)	
C3	0.14406 (14)	0.9941 (3)	0.23224 (17)	0.0308 (5)	
H3	0.1253	1.1003	0.1971	0.037*	
C4	0.21556 (14)	0.9938 (3)	0.3061 (2)	0.0409 (6)	
H4	0.2444	1.0989	0.3208	0.049*	
C5	0.24392 (14)	0.8370 (4)	0.3581 (2)	0.0450 (6)	
H5	0.2921	0.8361	0.4082	0.054*	
C6	0.20091 (13)	0.6821 (3)	0.33610 (17)	0.0361 (5)	
H6	0.2203	0.5772	0.3722	0.043*	
C7	0.08845 (12)	0.5008 (3)	0.24438 (16)	0.0225 (4)	
C8	0.02201 (11)	0.8642 (3)	0.13370 (14)	0.0204 (4)	

### Atomic displacement parameters (Å^2^)

**Table:**

	*U*^11^	*U*^22^	*U*^33^	*U*^12^	*U*^13^	*U*^23^
Cd1	0.03345 (14)	0.01501 (12)	0.01947 (13)	0.000	0.00909 (9)	0.000
Cd2	0.03771 (15)	0.01834 (13)	0.01698 (13)	0.00252 (8)	0.00830 (10)	−0.00369 (7)
O1	0.0559 (11)	0.0246 (8)	0.0209 (8)	−0.0100 (8)	0.0062 (7)	−0.0002 (6)
O1W	0.0392 (10)	0.0341 (10)	0.0287 (9)	0.0054 (7)	0.0116 (7)	0.0008 (7)
O2	0.0511 (10)	0.0304 (9)	0.0210 (7)	−0.0135 (8)	0.0105 (7)	0.0019 (6)
N1	0.0276 (9)	0.0179 (8)	0.0205 (9)	0.0017 (7)	0.0052 (7)	−0.0029 (6)
N2	0.0298 (10)	0.0247 (10)	0.0277 (10)	0.0045 (7)	0.0047 (8)	−0.0028 (7)
N3	0.0328 (10)	0.0261 (10)	0.0259 (9)	0.0037 (8)	0.0028 (8)	−0.0050 (8)
N4	0.0298 (9)	0.0218 (9)	0.0214 (9)	0.0027 (7)	0.0053 (7)	−0.0048 (7)
C1	0.0256 (10)	0.0215 (10)	0.0223 (10)	−0.0005 (8)	0.0095 (8)	−0.0014 (8)
C2	0.0256 (10)	0.0211 (10)	0.0204 (10)	−0.0008 (8)	0.0102 (8)	−0.0019 (8)
C3	0.0334 (13)	0.0233 (11)	0.0325 (13)	−0.0042 (9)	0.0113 (10)	0.0011 (8)
C4	0.0319 (13)	0.0332 (13)	0.0482 (16)	−0.0132 (10)	0.0090 (11)	−0.0041 (10)
C5	0.0262 (12)	0.0430 (14)	0.0456 (15)	−0.0062 (11)	−0.0029 (10)	0.0007 (12)
C6	0.0295 (12)	0.0306 (12)	0.0352 (13)	0.0011 (10)	0.0022 (10)	0.0063 (10)
C7	0.0242 (11)	0.0199 (10)	0.0212 (11)	0.0029 (7)	0.0077 (9)	0.0006 (7)
C8	0.0264 (10)	0.0156 (9)	0.0195 (9)	−0.0017 (8)	0.0102 (8)	−0.0010 (7)

### Geometric parameters (Å, °)

**Table:**

Cd1—N1^i^	2.2251 (17)	N1—N2	1.349 (3)
Cd1—N1^ii^	2.2251 (17)	N1—Cd1^v^	2.2251 (17)
Cd1—O2	2.2473 (16)	N2—N3	1.298 (3)
Cd1—O2^iii^	2.2473 (16)	N3—N4	1.347 (3)
Cd1—O1	2.6117 (17)	N4—C8	1.333 (3)
Cd1—O1^iii^	2.6118 (17)	C1—C6	1.390 (3)
Cd1—C7^iii^	2.779 (2)	C1—C2	1.404 (3)
Cd2—N4^iv^	2.2251 (18)	C1—C7	1.498 (3)
Cd2—N4	2.2251 (18)	C2—C3	1.394 (3)
Cd2—O1	2.2916 (16)	C2—C8	1.477 (3)
Cd2—O1^iv^	2.2916 (16)	C3—C4	1.378 (4)
Cd2—O1W	2.3623 (19)	C3—H3	0.9300
Cd2—O1W^iv^	2.3623 (19)	C4—C5	1.376 (4)
O1—C7	1.247 (3)	C4—H4	0.9300
O1W—H1W	0.75 (4)	C5—C6	1.375 (3)
O1W—H2W	0.87 (4)	C5—H5	0.9300
O2—C7	1.256 (3)	C6—H6	0.9300
N1—C8	1.337 (3)		
			
N1^i^—Cd1—N1^ii^	95.61 (9)	Cd2—O1—Cd1	127.14 (7)
N1^i^—Cd1—O2	128.32 (7)	Cd2—O1W—H1W	107 (3)
N1^ii^—Cd1—O2	105.23 (6)	Cd2—O1W—H2W	112 (2)
N1^i^—Cd1—O2^iii^	105.23 (6)	H1W—O1W—H2W	108 (4)
N1^ii^—Cd1—O2^iii^	128.32 (7)	C7—O2—Cd1	101.17 (13)
O2—Cd1—O2^iii^	97.83 (9)	C8—N1—N2	106.63 (16)
N1^i^—Cd1—O1	88.50 (6)	C8—N1—Cd1^v^	131.21 (14)
N1^ii^—Cd1—O1	151.13 (6)	N2—N1—Cd1^v^	121.56 (13)
O2—Cd1—O1	52.34 (5)	N3—N2—N1	108.79 (17)
O2^iii^—Cd1—O1	77.28 (6)	N2—N3—N4	109.07 (17)
N1^i^—Cd1—O1^iii^	151.13 (6)	C8—N4—N3	106.63 (17)
N1^ii^—Cd1—O1^iii^	88.50 (6)	C8—N4—Cd2	133.51 (14)
O2—Cd1—O1^iii^	77.28 (6)	N3—N4—Cd2	119.53 (13)
O2^iii^—Cd1—O1^iii^	52.34 (5)	C6—C1—C2	118.9 (2)
O1—Cd1—O1^iii^	101.55 (8)	C6—C1—C7	116.11 (19)
N1^i^—Cd1—C7^iii^	130.83 (7)	C2—C1—C7	125.01 (19)
N1^ii^—Cd1—C7^iii^	111.72 (7)	C3—C2—C1	118.60 (19)
O2—Cd1—C7^iii^	83.94 (7)	C3—C2—C8	115.22 (19)
O2^iii^—Cd1—C7^iii^	26.32 (6)	C1—C2—C8	126.00 (18)
O1—Cd1—C7^iii^	86.03 (6)	C4—C3—C2	121.5 (2)
O1^iii^—Cd1—C7^iii^	26.51 (6)	C4—C3—H3	119.2
N4^iv^—Cd2—N4	180.00 (10)	C2—C3—H3	119.3
N4^iv^—Cd2—O1	99.09 (6)	C5—C4—C3	119.6 (2)
N4—Cd2—O1	80.91 (6)	C5—C4—H4	120.2
N4^iv^—Cd2—O1^iv^	80.91 (6)	C3—C4—H4	120.2
N4—Cd2—O1^iv^	99.09 (6)	C6—C5—C4	119.9 (2)
O1—Cd2—O1^iv^	180.0	C6—C5—H5	120.1
N4^iv^—Cd2—O1W	91.33 (7)	C4—C5—H5	120.0
N4—Cd2—O1W	88.67 (7)	C5—C6—C1	121.5 (2)
O1—Cd2—O1W	93.94 (7)	C5—C6—H6	119.3
O1^iv^—Cd2—O1W	86.06 (7)	C1—C6—H6	119.2
N4^iv^—Cd2—O1W^iv^	88.67 (7)	O1—C7—O2	120.06 (19)
N4—Cd2—O1W^iv^	91.33 (7)	O1—C7—C1	122.33 (19)
O1—Cd2—O1W^iv^	86.06 (7)	O2—C7—C1	117.60 (19)
O1^iv^—Cd2—O1W^iv^	93.94 (7)	N4—C8—N1	108.88 (18)
O1W—Cd2—O1W^iv^	180.00 (9)	N4—C8—C2	129.86 (18)
C7—O1—Cd2	144.77 (14)	N1—C8—C2	120.98 (17)
C7—O1—Cd1	84.22 (13)		

Symmetry codes: (i) *x*, *y*−1, *z*; (ii) −*x*, *y*−1, −*z*+1/2; (iii) −*x*, *y*, −*z*+1/2; (iv) −*x*, −*y*+1, −*z*; (v) *x*, *y*+1, *z*.

### Hydrogen-bond geometry (Å, °)

**Table:**

*D*—H···*A*	*D*—H	H···*A*	*D*···*A*	*D*—H···*A*
O1W—H1W···O2^vi^	0.75 (4)	2.06 (4)	2.758 (2)	156 (4)
O1W—H2W···N2^vii^	0.87 (4)	2.14 (4)	2.961 (3)	155 (3)

Symmetry codes: (vi) *x*, −*y*+1, *z*−1/2; (vii) −*x*, −*y*+2, −*z*.
